# Distribution, toxicity load, and risk assessment of dissolved metal in surface and overlying water at the Xiangjiang River in southern China

**DOI:** 10.1038/s41598-020-80403-0

**Published:** 2021-01-08

**Authors:** Zhifeng Huang, Saisai Zheng, Yan Liu, Xingru Zhao, Xiaocui Qiao, Chengyou Liu, Binghui Zheng, Daqiang Yin

**Affiliations:** 1 National Engineering Laboratory for Lake Pollution Control and Ecological Restoration, State Environmental Protection Scientific Observation and Research Station for Lake Dongtinghu, Chinese Research Academy of Environmental Sciences, Beijing, 100012 China; 2 Key Laboratory of Yangtze River Water Environment, Ministry of Education, College of Environmental Science and Engineering, Tongji University, Shanghai, 200092 China; 3 Nanchang Institute of Technology, Nanchang, China

**Keywords:** Environmental monitoring, Environmental chemistry

## Abstract

Metal pollution in drinking water source has been under scrutiny as it seriously affects human health. This work examined 12 dissolved metals in the surface and overlying water of the Xiangjiang River, an important drinking water source in southern China, and characterized their distribution, identified their possible sources, assessed their toxicity load, and determined their potential ecological and health risk. No significant difference was found in the metal concentration between surface and overlying water. The average metal concentration fell in the order of Mg > Mn > Ba > Fe > Zn > As > Sb > Ni > Cd > V > Cr > Co, and all was lower than the safety threshold in the drinking water guideline of China. Anthropogenic activities were found to be the main source of metals from correlation analysis, principal component analysis (PCA), and cluster analysis (CA). According to the total heavy metal toxicity load (HMTL), 98.20%, 71.54%, 68.88%, and 7.97% of As, Cd, Sb, and Mn should be removed from the surface water to ensure safety. Most water samples from the surveyed area were found to have high ecological risk as was measured by the ecological risk index (RI). Health risk assessment showed that children are more susceptible than adults to the non-carcinogenic risk of dissolved metals, and the potential carcinogenic risk (CR) of As and Cd should be addressed. The results provide guidance for controlling the metal pollution of the Xiangjiang River and improving its quality as a drinking water source.

## Introduction

The quality of surface water is essential for the human society, as surface water supports not only residents in the urban environment but also the agriculture sector in the rural environment^1^. Surface water, mainly in the form of rivers and lakes, plays an irreplaceable role in urban development and human life. Urban rivers are an important sink of contaminants. As a result of industrialization and population growth, a large amount of contaminants, including trace metals, organic and inorganic compounds, etc., have been released into rivers and contaminated water^2^.

Metal contaminants in rivers are recognized for their persistence, environmental toxicity, bioaccumulation, etc.^3^. They may enter the human body directly from drinking water or indirectly via the food chain^4^. The metal contaminants in the water bodies may come from both natural sources (e.g., geological erosion, weathering, precipitation) and anthropogenic activities (e.g., mining, metal processing, industrial wastewater, the application of pesticides and fertilizers)^5–7^. In addition, sediments are also a source of metals because metals can be released into overlying water after desorption and then re-suspended in the surface water^8^.

Some metal elements are necessary for human metabolism (e.g., Cu, Zn, Fe, and Mn) but become toxic when their level exceeds a certain threshold. Some others have no physiological activity (e.g., As, Cd, Hg, and Pb) and damage the human's endocrine system, and are listed as environmental endocrine disruptors by the U.S. environmental protection agency (EPA)^9,10^. Many studies have illustrated the carcinogenic, teratogenic, and mutagenic effects of various trace metals. Therefore, it is of practical significance to investigate and assess the toxicity and health risk of metals in both surface water and overlying water.

The threat from metal contaminants in water bodies to human beings is particularly significant in developing countries including China. Over the past few decades, the Xiangjiang River has become one of the most heavily polluted rivers in China due to metallurgical industries and wastewater discharge from mining^11^. Many studies have examined metal pollution in the Xiangjiang River, which mainly focus on prevalent heavy metals (Zn, As, Cd, Cr, Ni, Co, etc.) but have largely overlooked metals such as Mg, V, Mn, Fe, Ba and Sb^12–14^. Meanwhile, many studies have investigated the concentration of metals in the Xiangjiang River and assessed the associated health risks but have not quantified the toxicity level of metals, and it remains unclear how much toxic metals must be removed before the water can become safe for human consumption^15^. To solve this problem, Saha and Paul developed in recent years a novel indicator, namely the heavy metal toxic load (HMTL)^16^. The use of HMTL can reliably estimate the toxicity load of metals in water and determine the required degree of removal for a given metal. Nevertheless, the use of HMTL is developed only recently and its application in existing literature is still limited^15,16^.

Systematic study is required to assess the distribution, possible sources, toxicity load, and the ecological and health risk of metals in surface and overlying water. In this work, we chose a typical area of the Xiangjiang River, and collected 60 water samples from the river to (1) characterize the distributions of 12 metals (Mg, V, Cr, Mn, Fe, Co, Ni, Zn, As, Cd, Sb, and Ba) in the water bodies, (2) identify possible sources of metal through principle component analysis (PCA), Pearson's correlation analysis, and cluster analysis, (3) assess the toxicity load of metal and determine by HMTL the necessary removal of the toxic metals from the water bodies, and (4) determine the potential ecological risk and health risk posed by the target metals in the river water. The current work is the first time that HMTL is applied to the assessment of metal toxic load of Chinese rivers. The results are expected to provide basic data and scientific evidence for the prevention and control of metal pollution in drinking water sources and help develop appropriate strategies for water quality management in nearby areas and similar riverine systems.

## Materials and methods

### Study area

The Xiangjiang River flows from south to north en route 6 major cities in Hunan, i.e., Yongzhou, Hengyang, Zhuzhou, Xiangtan, Changsha, and Yueyang, and finally joins the Yangtze River via the Dongting Lake. It is one of the main tributaries of the Yangtze River and a key drinking water source in southern China. The Xiangjiang river basin occupies 94,721 km^2^ (44.6% the area of the Hunan Province) and supports > 30 million residents of the Hunan Province^17^. The Hunan Province is known as the “Nonferrous Metal Village” because of its abundant mineral resources (e.g., Cd, Zn, Pb, Cu, etc.)^18^. However, the mining and smelting of nonferrous metals over the past years has caused severe metal pollution to the Xiangjiang River, especially in the Zhuzhou and Xiangtan sections^19^. These two cities are at the east of the Hunan Province and at the lower reaches of the Xiangjiang River with typical subtropical monsoon climate. The water depth is 1.3–10 m, and the average annual temperature is 16–18 °C. The average annual rainfall is approximately 1400 mm^13,20^.

### Sample collection and analysis

**Figure 1:**
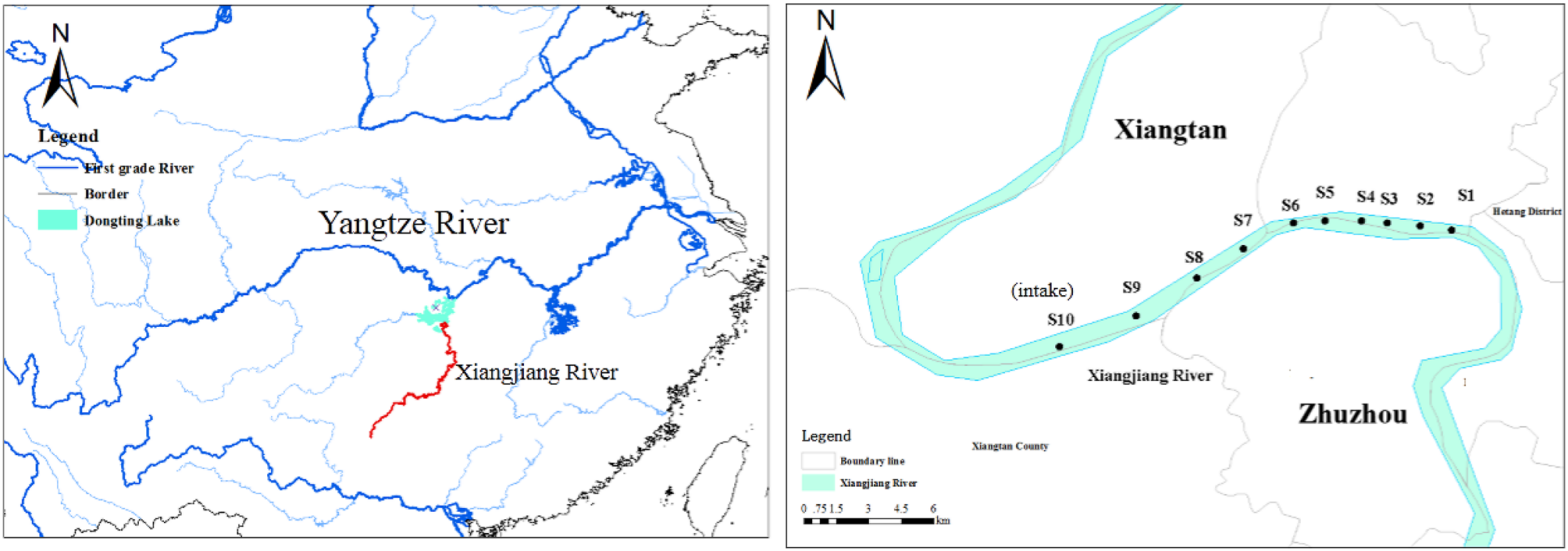
Map of the Xiangjiang River basin and the sampling sections.

To investigate the metal contamination status and the health risk of the drinking water source, the investigated area was divided into and represented by 10 sampling sections (S1–S10) from upstream to downstream (Fig. 1). The 10 sampling sections cover 2 cities: S1–S8 for Zhuzhou and S9 and S10 for Xiangtan. Specifically, S10 is located at the intake of a waterworks that provides service to urban residents. Detailed information about these sampling sections is presented in Table S1 of the Supplementary Information. In August 2011, 60 water samples in total were collected from a boat with a hydrophore at the study area, with 10 samples of surface water (0–15 cm from the river surface) and 10 samples of overlying water (10 cm above the river bottom) at the south, north (both within 0.5 m from the river bank), and middle (at the middle of the river) of each section, respectively. Water samples were taken in triplicates and collected in 1 L high-density polyethylene (HDPE) bottles. Two sets of water samples were collected simultaneously, with one used for physicochemical analysis (pH, TOC, TN) and the other for dissolved metal analysis. All water samples were stored in cooler boxes with ice packs before they were sent immediately to the laboratory for storage at 4 °C until further analysis.

Water samples were analyzed at the National Engineering Laboratory for Lake Pollution Control and Ecological Restoration of the Chinese Academy of Environmental Sciences. Total nitrogen (TN) was analyzed with a UV–Vis spectrophotometer (METASH UV-5300PC, Shanghai). Total organic carbon (TOC) was measured using a TOC analyzer (Shimazu, Japan). The pH value was measured using a digital meter (pHS-25, Leici Instrument Co., Shanghai). The concentrations of 12 dissolved metals (Mg, V, Cr, Mn, Fe, Co, Ni, Zn, As, Cd, Sb, and Ba) were analyzed by inductively coupled plasma mass spectrometry (ICP-MS) on an Agilent 7500 series instrument (USA). Prior to ICP-MS, water samples were filtered through a 0.45 μm micropore membrane (Whatman, USA) and acidified with 70% HNO_3_ until pH < 2, and then stored in HDPE bottle at 4 °C before metal analysis^21^.

### Heavy metal toxicity load (HMTL)

The heavy metal toxic load (HMTL) illustrates the degree of treatment required before water can become suitable for human use, and helps document effective treatment and management plans^15^. HMTL is derived by multiplying the concentration and the hazard intensity of a given heavy metal, as shown in Eq. (1):

1 $$\text{HMTL}={\sum }_{i=1}^{n}\text{C}\times \text{HIS}$$

where C is the concentration of heavy metal in water; *n* is the number of heavy metals, and HIS is the hazard intensity score. In this work, the HIS was assigned based on the occurrence frequency of the toxic metal as a harmful substance on the National Priorities List (NPL) maintained by the Agency for Toxic Substances and Disease Registry (ATSDR), the toxicity level of the studied metal, and the prospect of human contact^22^.

### Ecological risk assessment

The ecological risk index (RI) is a common measure to quantify the level of ecological risk of heavy metals in aquatic environment. It evaluates the potential damage from heavy metal contamination by the combined assessment of ecological risk and environmental toxicity^23^. The value of RI is calculated as follows:

2 $${{E}_{r}^{i}}{=}{{{T}}_{{r}}^{{i}}} \, \frac{{{\text{C}}_{\text{sample}}}}{{{\text{C}}_{\text{background}}}}$$

3 $$\text{RI} = \sum {{{E}}_{{r}}^{{i}}}$$

where C_sample_ and C_background_ are the measured concentration and background concentration, and ${{{E}}_{{r}}^{{i}}}$ and ${{T}}_{{r}}^{{i}}$ are the potential ecological risk factor and the toxic response factor of the heavy metal, respectively. The ${{T}}_{{r}}^{{i}}$ values for Cr, Mn, Co, Ni, Zn, As, Cd, and Sb are 2, 1, 5, 5, 1, 10, 30, and 10, respectively. The relationship between RI value and risk level is shown in Table S2 of the Supplementary Information.

### Human health risk assessment

In aquatic environmental pollution assessment, human health risk measured are calculated to evaluate the potential risk of water pollutants to human health by determining the intensity of pollutant exposure, the level of pollutant exposure, and the dose–response relationship between pollutants and human health. Human beings can be exposed to metals in river water through three pathways, namely direct ingestion, inhalation (through mouth and nose), and dermal absorption. Direct ingestion and dermal absorption are the main exposure pathways^24,25^. The average daily dose (ADD) into the human body by direct ingestion and dermal absorption was computed here according to Eqs. (4) and (5) based on the recommendations from the U.S. Environmental Protection Agency:

4 $${\text{ADD}}_{\text{ingestion}}=\frac{{\text{CW}} \times {\text{IR}} \times {\text{EF}} \times {\text{ED}}}{{\text{BW}} \times {\text{AT}}}$$

5 $${\text{ADD}}_{\text{dermal}} {=}\frac{{\text{CW}} \times {\text{SA}} \times {\text{IR}} \times {\text{EF}} \times {\text{ED}} \times {\text{ET}} \times {\text{K}}_{\text{p}} \times {10}^{-3}}{{\text{BW}} \times {\text{AT}}}$$

where ADD_ingestion_ and ADD_dermal_ indicate the average daily dose of exposure via ingestion and dermal adsorption (mg/kg/day), CW is the average concentration of the metal in water (μg/L), IR is the daily ingestion rate (L/day), EF is the exposure frequency (day/year), ED is the exposure duration (year), BW is the average body weight (kg), AT is the average time of exposure (days), Kp is the dermal permeability coefficient of the metal in water (cm/h), SA is the exposed skin area (cm^2^), and ET is the exposure time (h/day).

Both non-carcinogenic risk and carcinogenic risk were calculated for the studied metals. The non-carcinogenic risk, characterized by the hazard quotient (HQ), was calculated by dividing the average daily dose of the contaminant from each exposure pathway (ingestion, dermal) by the corresponding reference dose (RfD) by Eqs. (6) and (7):

6 $$ {\text{HQ }} = {\text{ ADD}}/{\text{RfD}} $$

7 $$ {\text{RfD}}_{{{\text{dermal}}}} = {\text{RfD}} \times {\text{ABS}}_{{{\text{GI}}}} $$

8 $${\text{HI}}=\sum_{\text{i=1}}^{\text{n}}{\text{HQ}}$$

where RfD can be retrieved from a risk-based concentration table. The comprehensive non-carcinogenic risk posed by the metals through the two exposure pathways was represented by the hazard index (HI) (Eq. (8)). Hazard to human health is likely when HQ > 1 or HI > 1^26^.

The carcinogenic risk (CR), which represents the probability of catching cancer during one’s lifetime as a result of carcinogenic exposure, was characterized by Eq. (9) ^25^.

9 $$ {\text{CR}} = {\text{ADD}} \times {\text{CSF}} $$

where CSF is the cancer slope factor (μg/kg/day)^−1^. The tolerable range of CR is advised by the U.S. EPA (2009) to be from 10^−6^ to 10^−4^. In this study, we only calculated the CR values for As, Cr, and Cd, which are the carcinogenic elements among the examined metals^25^. All listed parameters for the calculations are given in Tables S3 and S4 in the Supplementary Information.

### Statistical analysis

The Pearson correlation matrix and cluster analysis (CA) were formulated using SPSS 20.0 (SPSS Inc., Chicago, IL, USA) for correlation analysis to identify correlations among water quality parameters and metals in water samples. Principal component analysis (PCA) was performed using Canoco 5.0 (Biometris, Netherlands) to identify the source of the metals. Data analyses and statistical tests were performed using Origin 9 and Microsoft Excel 2010.

### Quality assurance and quality control (QA/QC)

Quality assurance and quality control (QA/QC) were implemented to ensure the accuracy of metal analysis. Three replicates were taken for the analysis of each parameter at each site. For dissolved metal analysis, blank samples were prepared to ensure that the chemicals used in the laboratory were not contaminated in any form. Procedural blank was analyzed after every 10 samples to verify accuracy. The recovery rate of metals was between 90 and 110%. Relative standard deviation was less than 10%. All containers prior to use for chemical analysis were immersed in 10% HNO_3_, stored for more than 24 h, and then washed successively with tap water and ultra-pure water.

## Results and discussion

### Distribution of metals

**Figure 2:**
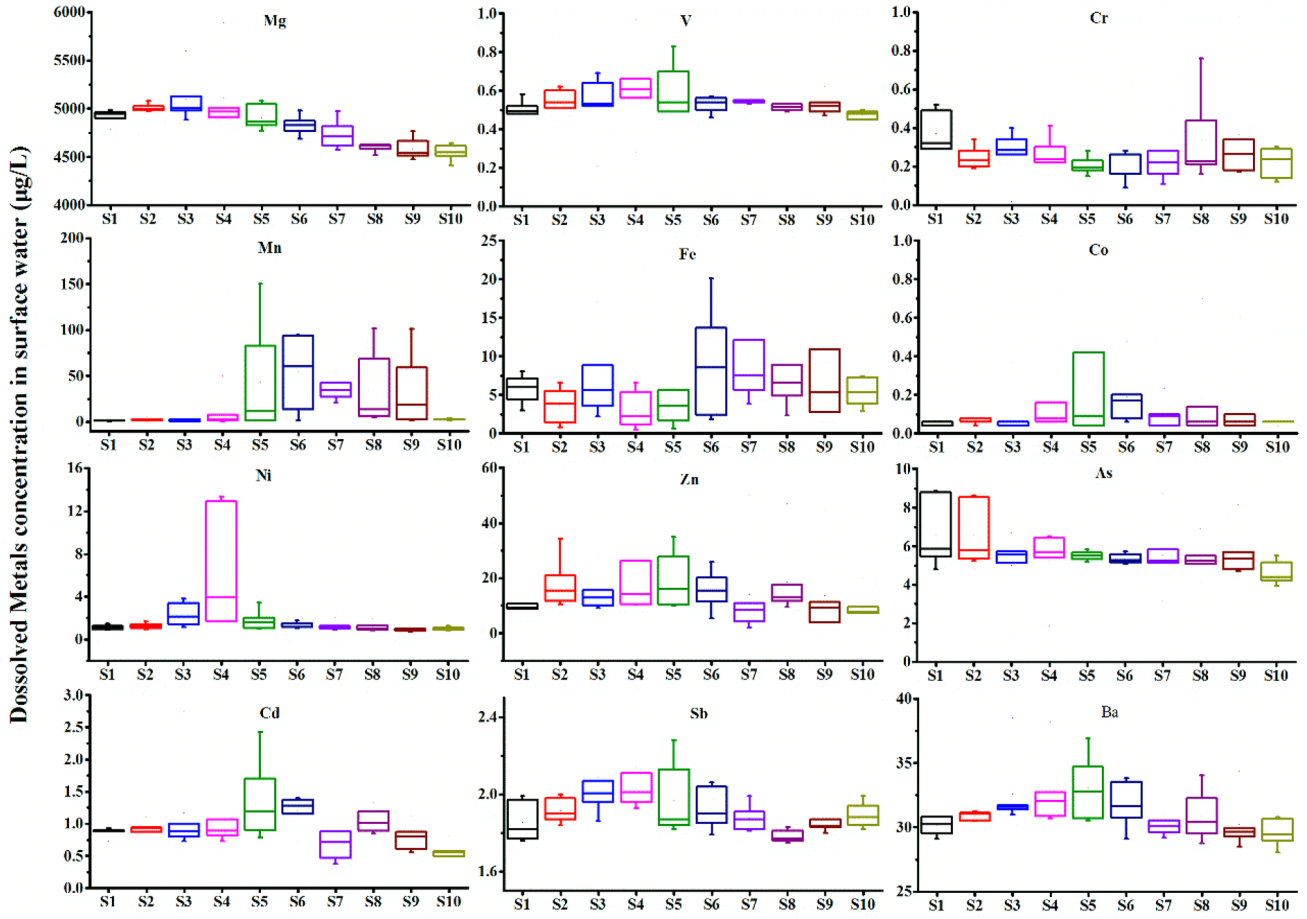
Spatial distribution of metals in surface water.

Table 1 summarizes the mean concentrations (μg/L) of the 12 dissolved metals in the surface water and overlying water of the Xiangjiang River. In the surface water samples, the concentration of Mg, V, Cr, Mn, Fe, Co, Ni, Zn, As, Cd, Sb, and Ba ranged in 4469.25–5889.31, 0.21–1.19, 0.02–0.98, 0.39–286.70, 0.64–432.30, 0.04–1.96, 0.88–13.27, 2.78–57.87, 3.95–8.86, 0.22–2.43, 1.79–2.8, and 29.10–38.17 μg/L, respectively (Fig. 2). The mean concentration of metal in surface water ranked in the order of Mg (4833.21 μg/L) > Mn (32.60 μg/L) > Ba (31.27 μg/L) > Fe (17.52 μg/L) > Zn (16.76 μg/L) > As (5.55 μg/L) > Sb (1.93 μg/L) > Ni (1.86 μg/L) > Cd (1.05 μg/L) > V (0.58 μg/L) > Cr (0.27 μg/L) > Co (0.19 μg/L). The average concentration of metals in the surface water was similar to that in the overlying water. The heavy metal concentrations (Cr, Zn, As, and Cd) found in the current study are significantly lower than the previous results in 2005 for the surface water of the Xiangjiang River, thus indicating a reduction of heavy metal pollution^27^. In addition, the newly determined heavy metal concentrations (Cr, Co, Ni, Zn, As, and Cd) are also significantly lower than the previously measured heavy metal concentrations in the sediments of the same area. This is because sediments are a reservoir of metals, and metals are deposited with suspended solids and accumulated in the sediments for a long time^28,29^.

**Table 1:** Concentration of metals in the Xiangjiang River and other rivers in southern China, and permissible limits of metals in drinking water (μg/L for metal in water, μg/g for metal in sediment).

	Mg	V	Cr	Mn	Fe	Co	Ni	Zn	As	Cd	Sb	Ba	Ref
Surface water in Xiangjiang River	4833.61	0.58	0.27	32.60	17.52	0.19	1.86	16.76	5.55	1.05	1.93	31.27	This study
Overlying water in Xiangjiang River	4862.06	0.55	0.25	28.80	8.67	0.17	1.77	16.34	5.50	1.03	1.94	31.42	
Surface water in Xiangjiang River (2005)			2.92					49.76	15.16	2.08			^27^
Sediment in Xiangjiang River (2011)			59.71			16.97	36.29	257.17	98.38	23.31			^34^
Yangtze River			4.54			0.07	1.35	9.53	3.05	0.09	1.19		^30^
Pearl River			1.70	1.06			1.89	3.61		0.04			^31^
Upper Han River		69.71	8.11	30.50	30.65	2.23	1.71		14.16	2.30	41.27	87.79	^32^
Southeastern hilly area rivers		1.10	2.05	45.59		0.11	2.19	32.45	2.02	0.30	1.22		^33^
**Water quality criteria for drinking water**													
China^a^		50	50	100	300		20	1000	10	5	5	700	
US EPA^b^			100					2000	10	5	6	2000	
WHO^c^	60,000		50	500	300	40	70	5000	10	3	20	2000	

^a^Chinese Ministry of Health 2007. Standards for drinking water quality (GB5749-2006).

^b^US EPA 2012. Edition of the drinking water standards and health advisories.

^c^WHO 2011. Guidelines for Drinking-water Quality.

The water quality of the Xiangjiang River is generally good. The mean concentration of all metals in both surface water and overlying water is lower than the permissible limit for drinking water specified by China (2007), US EPA (2012), and WHO (2011) (Table 1). Nevertheless, there is one surface water sample whose Fe concentration exceeds the permissible limit set by China and WHO. In addition, the Mn concentration of one surface water sample and one overlying water sample exceeded the permissible limits set by China but was lower than the permissible limit of WHO.

The Xiangjiang River is slightly more polluted by metals than other rivers in southern China (Table 1)^30–33^. Specifically, the Xiangjiang River has higher Co, Zn, As, and Cd concentration than the Yangtze River and the Pearl River, higher Mn and Ni concentration than the Upper Han River, and higher V and Sb concentration than all three rivers. These differences may be temporally specific and metal-specific^33^.

**Figure 3:**
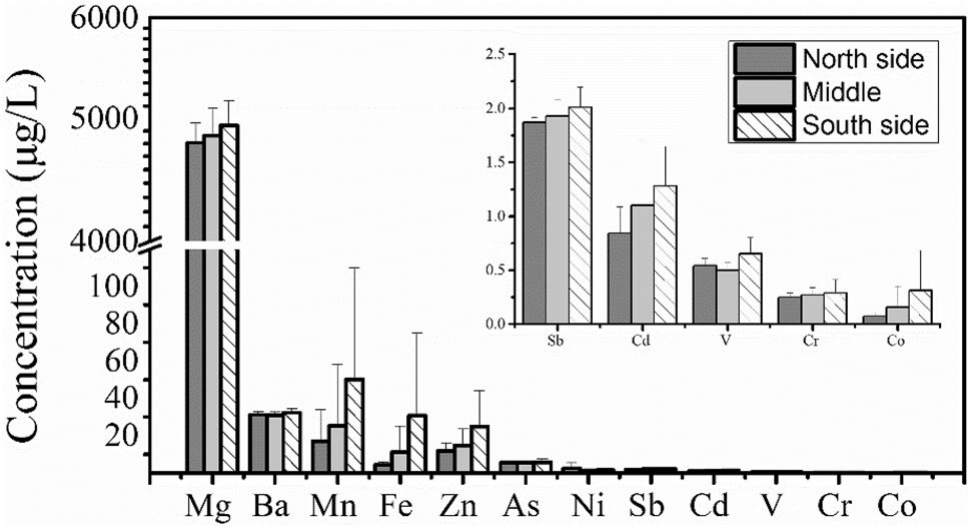
Metal concentrations in water samples collected at the south side, the north side, and in the middle of the river.

The metal concentrations in surface water have little spatial variation, and all metals except Cr and As show similar trends (Fig. 2). The metal concentration gradually increases along the river flow and peaks at midstream sections (S3–S6), then gradually decreases at downstream sections (S7–S10). Therefore, there may be point source pollution near the midstream of the study area. Several studies have been conducted on the local mining activities, and the development of agriculture has been suggested as the primary sources of metal pollution in the Xiangjiang River^14,17^. In addition, the concentration of metals is mostly significantly higher on the south side than on the north side or in the middle of the river. In particular, the concentration of Mn and Fe is nearly twice on the south side than on the north or in the middle (Fig. 3). This skewed distribution can be associated with the nonferrous metal mining and smelting plants in the south of the Xiangjiang River that produce Mn, Fe, and alloys and discharge wastewater. The spatial distribution of the metals in overlying water is similar, possibly because of the shallow water depth (average depth 5 m) in the studied area and full vertical mixing of pollutants (Supplementary Fig. S1).

### Identification of the sources of metals

Table 2 shows the Pearson correlation analysis among the metals in surface water and the physiochemical parameters of water. Significant positive correlation was observed among Mg, Mn, Co, Zn, Sb and Ba (*p* < 0.01), indicating homology and compound contamination to a great extent^35^. Significant positive correlation was also found among V, Cd, Fe, and As, with coefficients ranging in 0.39–0.96 (*p* < 0.01 or *p* < 0.05), indicating similar sources for these metals. Ni is not related to any metal except Mg, thus indicating a distinct source for Ni. Some environmental factors also appear to affect the distribution of metals. In the current results, pH and TOC were not the main factors affecting the studied metals in the Xiangjiang River water, whereas significant positive correlations was found between TN and most metals (except Mg, Fe, and Ni), indicating that the TN and the metals have common sources and identical impact on the water environment.

**Table 2:** Pearson correlation coefficient (*r*) among metals in the surface water of the Xiangjiang River and the physiochemical parameters of the river.

	Mg	V	Cr	Mn	Fe	Co	Ni	Zn	As	Cd	Sb	Ba	pH	TOC	TN
Mg	1	0.05	− 0.12	0.61**	0.08	0.69**	0.38*	0.61**	− 0.09	0.32	0.83**	0.69**	− 0.31	− 0.27	− 0.06
V		1	0.76**	0.36	0.96**	0.34	0.06	0.50**	0.61**	0.77**	− 0.14	0.47**	− 0.06	0.24	0.86**
Cr			1	0.18	0.74**	0.16	− 0.08	0.35	0.70**	0.70**	− 0.29	0.26	0.07	0.23	0.80**
Mn				1	0.49**	0.97**	0.18	0.83**	− 0.17	0.67**	0.69**	0.85**	− 0.1	0.05	0.46**
Fe					1	0.47**	0.02	0.57**	0.50**	0.77**	− 0.08	0.48**	− 0.06	0.31	0.89**
Co						1	0.22	0.85**	− 0.17	0.64**	0.75**	0.85**	− 0.15	0.04	0.42*
Ni							1	0.14	− 0.05	0.08	0.35	0.37*	− 0.27	− 0.23	0.01
Zn								1	0.06	0.82**	0.55**	0.82**	− 0.12	0.05	0.58**
As									1	0.39*	− 0.33	0.03	0.00	0.19	0.43*
Cd										1	0.18	0.73**	0.11	0.08	0.82**
Sb											1	0.67**	− 0.29	− 0.22	− 0.14
Ba												1	− 0.2	− 0.22	0.48**
pH													1	− 0.03	− 0.31
TOC														1	0.26
TN															1

Statistically significant values are shown in bold.

**Extremely significant correlation, *p* < 0.01 (2-tailed).

*Significant correlation, *p* < 0.05 (2-tailed).

Principle component analysis (PCA) with varimax rotation was further performed to identify the possible sources of metals based on the determined metal concentration (Table 3). PCA is used to reduce the dimensionality of variables because the KMO test gives > 0.7 and the Bartlett test gives *p* < 0.001^36^. Three principal components (factor 1, factor 2, and factor 3) with eigenvalue > 1 were extracted. These three factors explained 86.77% of the overall variance, with 43.07% by factor 1, 33.34% by factor 2, and 10.36% by factor 3. The factor loading is referred to as “strong”, “moderate”, and “weak” when the loading value is > 0.75, 0.75–0.50, and 0.50–0.30, respectively^37^. Factor 1 has strong positive loading for Mg, Mn, Co, Zn, Sb, and Ba, moderate positive loading for Cd, and weak positive loading for Fe. Factor 2 has strong positive loading for V, Cr, Fe, Cd, and As, and weak positive loading for Zn and Ba. Factor 3 has strong positive loading for Ni and weak positive loading for Mg and Sb. The principal components 1 and 2 are likely from the anthropogenic activities including mining, metal processing, and industrial or agricultural wastewater. The co-occurrence of Mg, Mn, Zn, Sb, Co, and Ba may have resulted from voluminous wastewater from many industrial enterprises along the Xiangjiang River^17,38^, and Zn, Mn and Sb may have originated from mining processes as a result of the rapidly developing mining industry in the Hunan province. In contrast, Cr and Fe may have come from industrial wastewater discharged by various industrial processes such as electroplating and alloy processes^20^. Natural activities may have mainly contributed to the metal pollution by factor 3, which is dominated by Ni and explains relatively little (10.36%) of the total variance. Previous studies of the Zijiang River in southern China have also found Ni to come from natural source^39^.

**Table 3:** Principal component analysis for dissolved metals.

	Factor 1	Factor 2	Factor 3
Mg	0.75	− 0.11	0.44
V	0.22	0.92	0.00
Cr	0.02	0.91	− 0.09
Mn	0.95	0.16	− 0.04
Fe	0.33	0.87	− 0.10
Co	0.97	0.14	0.02
Ni	0.17	0.00	0.91
Zn	0.86	0.37	− 0.02
As	− 0.28	0.80	0.18
Cd	0.59	0.73	− 0.05
Sb	0.81	− 0.34	0.32
Ba	0.86	0.30	0.26
Total	5.17	4.00	1.24
% of variance	43.07	33.34	10.36
Cumulative%	43.07	76.41	86.77

**Figure 4:**
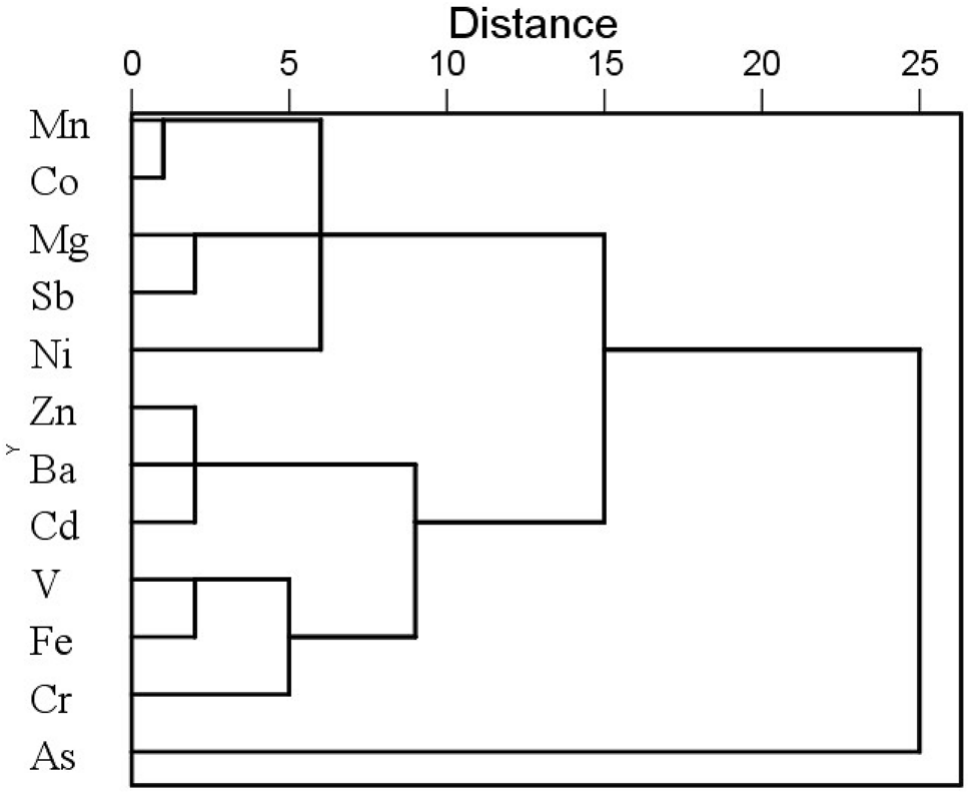
Cluster analysis dendrogram of metals in surface water.

Cluster analysis (CA) was applied to segment the metals by Ward’s method as shown in the dendrogram (Fig. 4), in which the metals in surface water were clustered into 3 categories: (1) Mn, Co, Mg, Sb, and Ni, (2) Zn, Ba, Cd, V, Fe, and Cr, and (3) As. Smaller distance in the dendrogram indicates closer relationship between the elements. Compared with the PCA result on metal source classification, the CA result is reasonably similar to and few metals appear to be outliers.

### Heavy metal toxicity load (HMTL) in water

Heavy metal toxicity load (HMTL) can evaluate the level of toxic metals in water bodies and indicate the needed removal of toxic metals to make the water safe for human use^16^. The HMTL index determines the toxicity level of pollutant in water that results in non-carcinogenic risk (Kumar et al.) and helps in providing an efficient treatment and management plan^40^. The HMTL was calculated for the toxic metals Cr, Mn, Co, Ni, Zn, As, Cd, Sb and Ba, all of which were selected from the ATSDR substance priority list (ATSDR)^41^. The permissible concentration (mg/L) of the metals was as follows: Cr, 0.05; Mn, 0.3; Co, 0.002; Ni, 0.07; Zn, 5; As, 0.001; Cd, 0.003; Sb, 0.006; Ba, 2 (ATSDR 2018)^42^. The HMTL of metals ranged from 44,921.4 to 105,478.6 mg/L in the surface water with a mean of 80,431.1 mg/L (Table 4), and ranged from 22,489.2 to 57,781.1 mg/L in the overlying water with a mean of 42,208.2 mg/L (Supplementary Table S5). The HTML in the present study is lower than the permissible toxicity load, indicating a low level of contamination of toxic metals in water. However, continuous water pollution may further increase the HMTL. According to the calculated total HMTL, 7.97%, 98.20%, 71.54%, and 68.88% of Mn, As, Cd, and Sb in the surface water and 98.18%, 70.78%, and 69.08% of As, Cd, and Sb in the overlying water need to be eliminated from the Xiangjiang River (Table 4, Supplementary Table S5). Removal of the excess metals from the water body is necessary to address safety and health concerns.

**Table 4:** Heavy metal toxicity load (HMTL, μg/L) of the surface water based on the relative level of heavy metals.

Surface water	Toxicity of heavy metals (μg/L)									
	Cr	Mn	Co	Ni	Zn	As	Cd	Sb	Ba	HMTL
S1	331.9	933.8	47.2	1110.5	8226.1	11,067.2	1144.5	1114.9	24,101.3	48,077.4
S2	218.8	2136.0	74.1	1256.1	16,449.2	11,000.1	1241.1	1150.9	24,722.7	58,249.1
S3	236.6	39,413.0	374.1	2317.0	25,816.6	8430.3	1544.3	1256.1	26,090.7	105,478.6
S4	211.3	39,916.4	394.3	6209.6	20,229.0	8829.7	1329.0	1284.1	26,200.0	104,603.5
S5	183.1	34,421.1	185.4	1762.6	17,459.6	9243.1	1799.1	1183.0	26,452.0	92,688.9
S6	165.2	43,139.0	195.5	1395.2	14,233.7	8966.6	1581.6	1155.9	25,381.3	96,213.9
S7	275.3	43,301.0	235.9	1209.8	12,737.9	9329.7	1320.2	1128.9	24,688.0	94,226.7
S8	300.6	27,731.6	175.2	1165.1	16,972.7	8969.4	1755.1	1070.8	24,721.3	82,861.9
S9	327.4	26,686.2	151.7	1041.0	12,509.6	9505.7	1471.8	1105.8	24,190.7	76,989.9
S10	196.5	2122.7	57.3	1001.3	8366.1	7704.0	707.3	1136.9	23,629.3	44,921.4
Total	2446.8	259,800.7	1890.6	18,468.1	153,000.5	93,045.9	13,893.9	11,587.3	250,177.3	804,311.3
Hazard intensity scere (HIS)^a^	893	797	1011	993	913	1676	1318	601	800	
Permissible toxicity load (μg/L)	44,650	239,100	2022	69,510	4,565,000	1676	3954	3606	1,600,000	
Removal of toxic metal to reduce pollution load	PTL	7.97%	PTL	PTL	PTL	98.20%	71.54%	68.88%	PTL	

*PTL* within permissible toxicity load.

^a^ATSDR 2019.

### Ecological risk index (RI)

**Figure 5:**
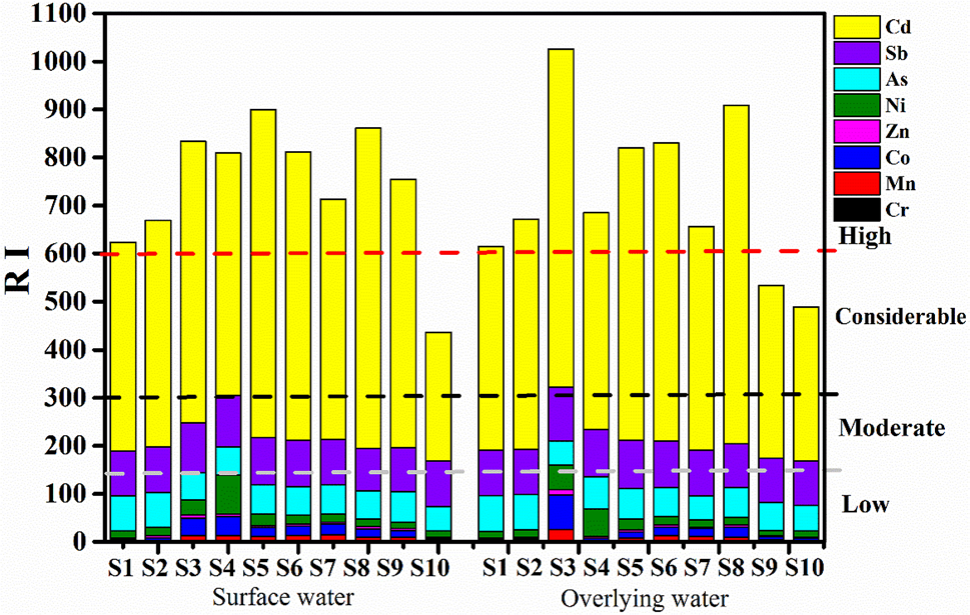
RI values of heavy metals in the water samples.

Most sampling sections in the surveyed area have high ecological risk from heavy metal pollution (Fig. 5). The calculated ecological risk index (RI) of the heavy metals ranged from 436.38 to 899.92 with a mean value of 741.29 in surface water, and from 489.00 to 1025.84 with a mean of 723.44 in overlying water. With respect to the risk index of a single element (${{{E}}_{{r}}^{{i}}}$), Cd incurred the highest ecological risk at all sections (${{{E}}_{{r}}^{{i}}}$ > 320), and Sb (80 ≤ ${{{E}}_{{r}}^{{i}}}$< 160) and As (40 ≤${{{E}}_{{r}}^{{i}}}$  < 80) also created considerable ecological risk at all sections. Other heavy metals (Ni, Zn, Co, Mn, and Cr) induced lower ecological risk (${{{E}}_{{r}}^{{i}}}$ < 40).

### Health risk assessment analysis

**Figure 6:**
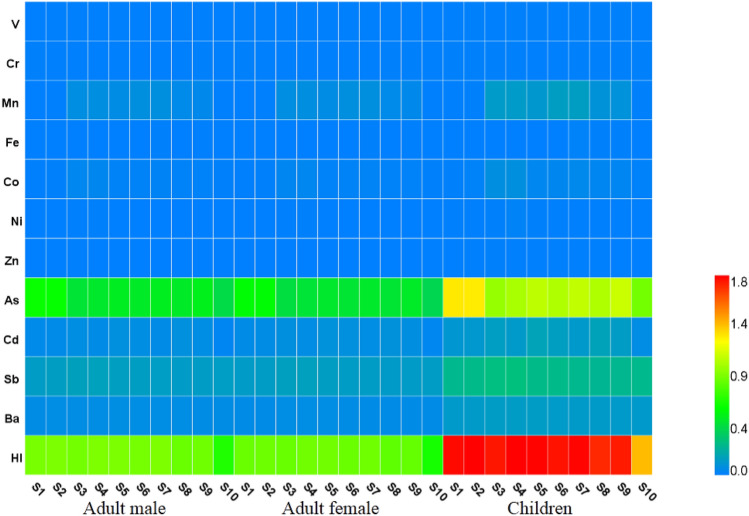
HQ and HI values of metals in surface water.

The health risk assessment of metals in drinking water sources has been receiving extensive attention because the quality of drinking water has a strong influence on the wellbeing of the affected population^43–45^. Figure 6 shows for each metal the HQ and HI values in surface and overlying water. Supplementary Tables S6–S8 present the calculated HQ value of each metal for adult male, female, and children. With regard to non-carcinogenic risks, the HI of metals in surface water ranged from 0.71 to 0.95 for adult male, from 0.69 to 0.91 for adult female, and from 1.44 to 1.90 for children. That is, no non-carcinogenic risk is found for adults from exposure to metals in the Xiangjiang River because all determined HI and HQ values are less than 1 (Fig. 6). However, As in surface water shows high HQ value (> 1) for children, which further results in an HI value higher than 1 in all water samples (Fig. 6). That is, exposure to As is a potential non-carcinogenic risk to local children. This finding is consistent with other studies on various rivers in southern China and of the Three Gorges Reservoir^33,46^. The health risk differs notably depending on the pathway of intake (i.e., ingestion or dermal absorption). The average HI value of ingestion is 1–3 orders of magnitude greater than that of dermal absorption (Supplementary Tables S6–S8), indicating that ingestion is the primary exposure that generates health risk^47^. Children are more susceptible to As than adults as the mean total health risk (HI) of As is about 2.2 times for children than for adults, which can be attributed to the differences of physiological and behavioral characteristics between children and adults (EPA, 2008). Children are more sensitive to external environment during their growth and development^48^.

**Figure 7:**
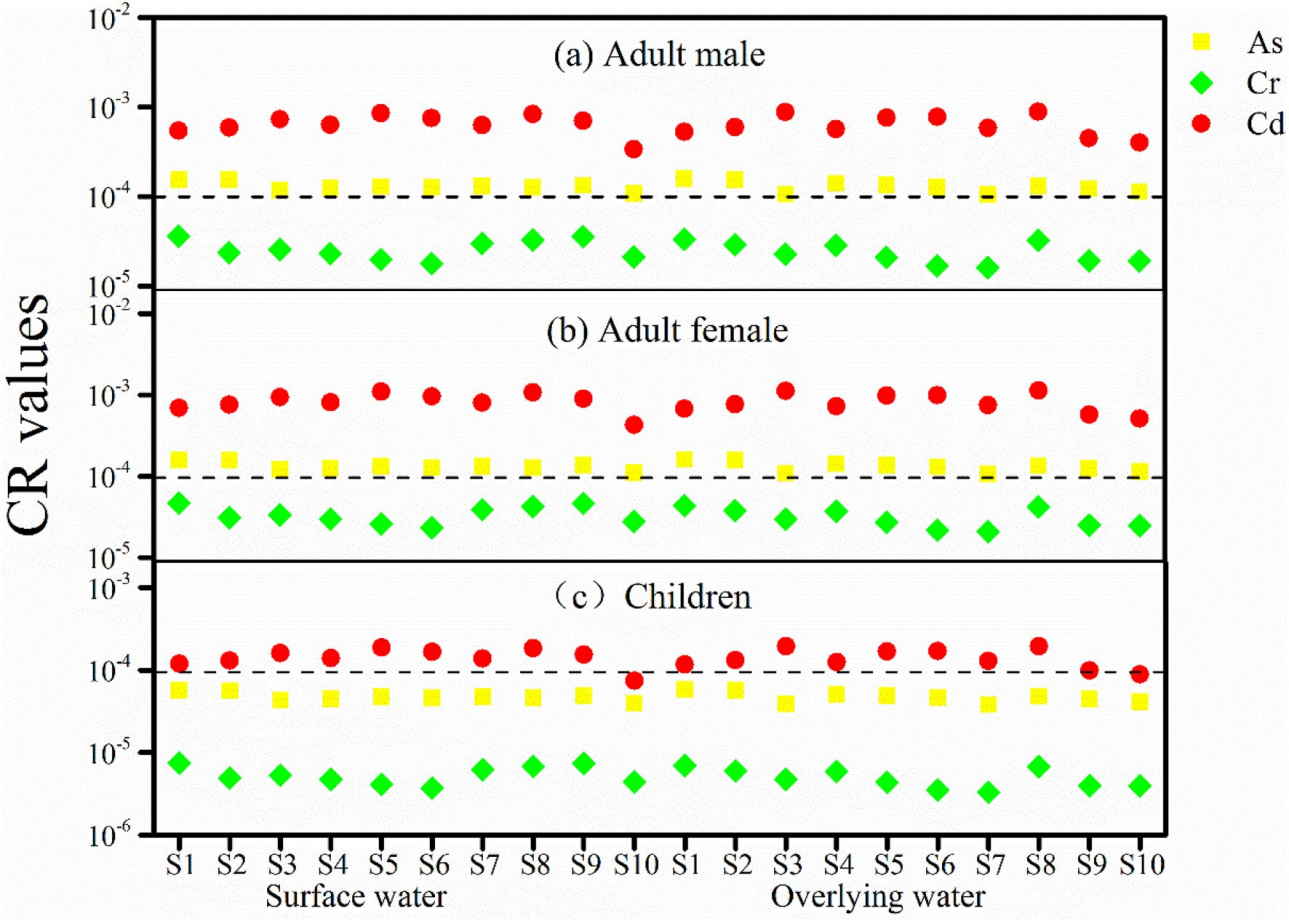
CR values of metals in the Xiangjiang River for (**a**) adult male, (**b**) adult female, and (**c**) children.

The CR values of As, Cr, and Cd were assessed for adult male, adult female, children to quantify the carcinogenic risk (Fig. 7). Exposure to Cr at the current level does not show carcinogenic risk to either adult or children (CR < 10^−4^). However, carcinogenic risk from Cd and As can be recognized for all water samples as the CR values all breach or approach the safety limit (10^−4^). The CR value of As is 1.30 × 10^−4^, 1.33 × 10^−4^, and 4.76 × 10^−5^, and the CR value of Cd is 6.61 × 10^−4^, 8.46 × 10^−4^, and 1.45 × 10^−4^ for adult male, adult female, and children, respectively (Fig. 7). Therefore, among the heavy metals in the Xiangjiang River, Cd and As are the major sources of carcinogenic risk. Previous studies have also found carcinogenic risks from exposure to arsenic and cadmium in surface water^49,50^. It has been shown that As in drinking water can cause liver cancer, lung cancer, hypertension, neuropathy, etc., and Cd can damage lung and trigger DNA mutation^24,51^. Effective measures are needed to manage metal pollution in the Xiangjiang River to reduce the level of Cd and As in the drinking water source, protect human health, and ensure the healthy development of the aquatic ecosystem.

It is worth noting that in this study, the health risk assessment of metal is a deterministic process that uses reasonable maximum exposure parameters to obtain conservative results. In addition, some previous studies showed seasonal differences in metal concentration, i.e., lower in summer than winter due to rainwater dilution^15,33^. Since the samples in this study were taken in summer, underestimation of the associated risk may be possible. Thus, future work plans to adopt methods such as probabilistic risk assessment (PRA) to address these uncertainty by calculating risks based on the range and statistical distribution of exposure parameters^52,53^.

## Conclusions

This study investigated 12 metals in the Xiangjiang River in southeastern China with focus on their pollution characteristics, toxicity load, and risk assessment. The presence of toxic metals in surface and overlying water was found to have an adverse effect on ecological environment and human health. Correlation analysis, principle component analysis (PCA), and cluster analysis (CA) were applied to identify the source of metal pollution in the Xiangjiang River. Most metals come from anthropogenic activities including mineral exploitation and industrial wastewater, and Ni can be attributed to natural sources. The metals As, Cd, Sb, and Mn in the Xiangjiang River have higher heavy metal toxicity load (HMTL) than the permissible limit. Therefore, toxic metals need to be removed for the Xiangjiang River to be a safe source of drinking water. According to ecological risk index (RI), there is high ecological risk from the studied metals (RI > 600). The metals pose potential non-carcinogenic and carcinogenic risk (CR) to adult male, adult female, and children through ingestion and dermal absorption, and children are more susceptible to the non-carcinogenic risk of dissolved metals. Cd and As may incur potential carcinogenic risk (CR > 10^−4^) to the affected population and should be dealt with seriously.

## Supplementary Information

Supplementary Information.

## Data Availability

The datasets obtained and analyzed in the current study are available from the corresponding author upon request.

## References

[1] Brack W (2017). Towards the review of the European Union Water Framework Directive: Recommendations for more efficient assessment and management of chemical contamination in European surface water resources. Sci. Total Environ..

[2] Su S (2013). Spatial determinants of hazardous chemicals in surface water of Qiantang River, China. Ecol. Indic..

[3] Zhang C (2014). Effects of sediment geochemical properties on heavy metal bioavailability. Environ. Int..

[4] Zhang D (2012). Distribution of heavy metals in water, suspended particulate matter and sediment of Poyang lake, China. Fresenius Environ. Bull..

[5] Wang X, Han J, Xu L, Gao J, Zhang Q (2011). Effects of anthropogenic activities on chemical contamination within the Grand Canal, China. Environ. Monit. Assess..

[6] Botelho RG, Rossi ML, Maranho LA, Olinda RA, Tornisielo VL (2013). Evaluation of surface water quality using an ecotoxicological approach: A case study of the Piracicaba River (So Paulo, Brazil). Environ. Sci. Pollut. Res..

[7] Zeng Y (2019). Health risk assessment and source apportionment for heavy metals in a southern chinese reservoir Impacted by stone mining activities. Integr. Environ. Assess. Manage..

[8] Liang J, Liu J, Xu G, Chen B (2019). Distribution and transport of heavy metals in surface sediments of the Zhejiang nearshore area, East China Sea: Sedimentary environmental effects. Mar. Pollut. Bull..

[9] Graedel TE (2005). The multilevel cycle of anthropogenic zinc. J. Ind. Ecol..

[10] Bhattacharya PT, Misra SR, Hussain M (2016). Nutritional aspects of essential trace elements in oral health and disease: An extensive review. Scientifica.

[11] Chen J-Q, Wang Z-X, Wu X, Zhu J-J, Zhou W-B (2011). Source and hazard identification of heavy metals in soils of Changsha based on TIN model and direct exposure method. Trans. Nonferrous Met. Soc. China.

[12] Li H, Yang J, Ye B, Jiang D (2019). Pollution characteristics and ecological risk assessment of 11 unheeded metals in sediments of the Chinese Xiangjiang River. Environ. Geochem. Health.

[13] Jiang D (2019). Multivariate analyses and human health assessments of heavy metals for surface water quality in the Xiangjiang River Basin, China. Environ. Toxicol. Chem..

[14] Fang X (2019). Distribution, contamination and source identification of heavy metals in bed sediments from the lower reaches of the Xiangjiang River in Hunan province, China. Sci. Total Environ..

[15] Proshad R (2020). Appraisal of heavy metal toxicity in surface water with human health risk by a novel approach: A study on an urban river in vicinity to industrial areas of Bangladesh. Toxin Rev..

[16] Saha P, Paul B (2019). Assessment of heavy metal toxicity related with human health risk in the surface water of an industrialized area by a novel technique. Hum. Ecol. Risk Assess..

[17] Chai L (2017). Heavy metals and metalloids in the surface sediments of the Xiangjiang River, Hunan, China: Distribution, contamination, and ecological risk assessment. Environ. Sci. Pollut. Res..

[18] Peng B (2011). Geochemistry of trace metals and Pb isotopes of sediments from the lowermost Xiangjiang River, Hunan Province (P. R. China): Implications on sources of trace metals. Environ. Earth Sci..

[19] Zhang Q (2009). Assessment of surface water quality using multivariate statistical techniques in red soil hilly region: A case study of Xiangjiang watershed, China. Environ. Monit. Assess..

[20] Zeng X (2015). Spatial distribution, health risk assessment and statistical source identification of the trace elements in surface water from the Xiangjiang River, China. Environ. Sci. Pollut. Res..

[21] Liu H, Li W (2011). Dissolved trace elements and heavy metals from the shallow lakes in the middle and lower reaches of the Yangtze River region, China. Environ. Earth Sci..

[22] APHA (2012). Standard Methods for Examination of Water and Waste Water.

[23] Proshad R, Kormoker T, Islam S (2019). Distribution, source identification, ecological and health risks of heavy metals in surface sediments of the Rupsa River, Bangladesh. Toxin Rev..

[24] Wu B (2009). Preliminary risk assessment of trace metal pollution in surface water from yangtze river in Nanjing section, China. Bull. Environ. Contam. Toxicol..

[25] Li S, Zhang Q (2010). Spatial characterization of dissolved trace elements and heavy metals in the upper Han River (China) using multivariate statistical techniques. J. Hazard. Mater..

[26] Yang M, Fei Y, Ju Y, Ma Z, Li H (2012). Health risk assessment of groundwater pollution—A case study of typical city in North China plain. J. Earth Sci..

[27] Jiang D (2019). Multivariate analyses and human health assessments of heavy metals for surface water quality in the Xiangjiang river basin, China. Environ. Toxicol. Chem..

[28] Islam MS, Ahmed MK, Raknuzzaman M, Habibullah-Al-Mamun M, Islam MK (2015). Heavy metal pollution in surface water and sediment: A preliminary assessment of an urban river in a developing country. Ecol. Ind..

[29] Ali MM, Ali ML, Islam MS, Rahman MZ (2016). Preliminary assessment of heavy metals in water and sediment of Karnaphuli River, Bangladesh. Environ. Nanotechnol. Monit. Manage..

[30] Yin S (2016). Contribution of the upper river, the estuarine region, and the adjacent sea to the heavy metal pollution in the Yangtze Estuary. Chemosphere.

[31] Geng J, Wang Y, Luo H (2015). Distribution, sources, and fluxes of heavy metals in the Pearl River Delta, South China. Mar. Pollut. Bull..

[32] Li S, Zhang Q (2010). Risk assessment and seasonal variations of dissolved trace elements and heavy metals in the Upper Han River, China. J. Hazard. Mater..

[33] Xu J (2020). Identification of dissolved metal contamination of major rivers in the southeastern hilly area, China: Distribution, source apportionment, and health risk assessment. Environ. Sci. Pollut. Res. Int..

[34] Huang Z, Liu C, Zhao X, Dong J, Zheng B (2020). Risk assessment of heavy metals in the surface sediment at the drinking water source of the Xiangjiang River in South China. Environ. Sci. Eur..

[35] Song Y (2011). Geochemical behavior assessment and apportionment of heavy metal contaminants in the bottom sediments of lower reach of Changjiang River. CATENA.

[36] Zhou F, Guo H, Liu L (2007). Quantitative identification and source apportionment of anthropogenic heavy metals in marine sediment of Hong Kong. Environ. Geol..

[37] Wang J, Liu G, Liu H, Lam PKS (2017). Multivariate statistical evaluation of dissolved trace elements and a water quality assessment in the middle reaches of Huaihe River, Anhui, China. Sci. Total Environ..

[38] Li F (2013). Spatial risk assessment and sources identification of heavy metals in surface sediments from the Dongting Lake, Middle China. J. Geochem. Explor..

[39] Zhang Z (2018). Assessment of heavy metal contamination, distribution and source identification in the sediments from the Zijiang River, China. Sci. Total Environ..

[40] Kumar, V. *et al.* Global evaluation of heavy metal content in surface water bodies: A meta-analysis using heavy metal pollution indices and multivariate statistical analyses. *Chemosphere* **236**. 10.1016/j.chemosphere.2019.124364 (2019). 10.1016/j.chemosphere.2019.124364 31326755

[41] ATSDR (Agency for Toxic Substances and Disease Registry). *Agency for Toxic Substances and Disease Registry, Substance Priority List*. https://www.atsdr.cdc.gov/spl/. Accessed 19 Aug 2020 (2019).

[42] ATSDR. *Toxic Substance Portal: Toxicological Profiles*. https://www.atsdr.cdc.gov/toxprofiles/index.asp. Accessed 19 Aug 2020 (2018).

[43] Wang R-S, Xu Q-J, Zhang X, Wei Q-S, Yan C-Z (2012). Health risk assessment of heavy metals in typical township water sources in DongjianG River basin. Huanjing Kexue.

[44] Yi Y, Yang Z, Zhang S (2011). Ecological risk assessment of heavy metals in sediment and human health risk assessment of heavy metals in fishes in the middle and lower reaches of the Yangtze River basin. Environ. Pollut..

[45] Zhao YW, Zhou LQ, Dong BQ, Dai C (2019). Health assessment for urban rivers based on the pressure, state and response framework—A case study of the Shiwuli River. Ecol. Ind..

[46] Zhao L (2020). Spatial-temporal distribution characteristics and health risk assessment of heavy metals in surface water of the Three Gorges Reservoir, China. Sci. Total Environ..

[47] Kerger BD, Paustenbach DJ, Corbett GE, Finley BL (1996). Absorption and elimination of trivalent and hexavalent chromium in humans following ingestion of a bolus dose in drinking water. Toxicol. Appl. Pharmacol..

[48] Bartrem C (2014). Unknown risk: Co-exposure to lead and other heavy metals among children living in small-scale mining communities in Zamfara State, Nigeria. Int. J. Environ. Health Res..

[49] Liang B (2018). Distribution, sources, and water quality assessment of dissolved heavy metals in the Jiulongjiang River Water, southeast China. Int. J. Environ. Res. Public Health..

[50] Emenike PC, Neris JB, Tenebe IT, Nnaji CC, Jarvis P (2020). Estimation of some trace metal pollutants in River Atuwara southwestern Nigeria and spatio-temporal human health risks assessment. Chemosphere.

[51] Zohra BS, Habib A (2016). Assessment of heavy metal contamination levels and toxicity in sediments and fishes from the Mediterranean Sea (southern coast of Sfax, Tunisia). Environ. Sci. Pollut. Res..

[52] Junaid M, Hashmi MZ, Tang Y-M, Malik RN, Pei D-S (2017). Potential health risk of heavy metals in the leather manufacturing industries in Sialkot, Pakistan. Sci. Rep..

[53] Peng C, Cai Y, Wang T, Xiao R, Chen W (2016). Regional probabilistic risk assessment of heavy metals in different environmental media and land uses: An urbanization-affected drinking water supply area. Sci. Rep..

