# Supplementary material for: Distribution, toxicity load, and risk assessment of dissolved metal in surface and overlying water at the Xiangjiang River in southern China
Source: Sci Rep. 2021 Jan 8;11:109. doi: 10.1038/s41598-020-80403-0 (PMC7794442; doi:10.1038/s41598-020-80403-0)
Supplement: Supplementary file 1 — Supplementary Information. [file 41598_2020_80403_MOESM1_ESM.pdf]

Suppplementary Material

Distribution, Toxicity Load, and Risk Assessment of Dissolved Metal in Surface and Overlying Water at the Xiangjiang River in Southern China

Zhifeng Huang[1,2], Saisai Zheng[3], Yan Liu[1], Xingru Zhao[1]*, Xiaocui Qiao[1], Chengyou Liu[1], Binghui Zheng[1]*, Daqiang Yin[2]

[1] National Engineering Laboratory for Lake Pollution Control and Ecological Restoration, State Environmental Protection Scientific Observation and Research Station for Lake Dongtinghu, Chinese Research Academy of Environmental Sciences, Beijing 100012, China.
[2] Key Laboratory of Yangtze River Water Environment, Ministry of Education, College of Environmental Science and Engineering, Tongji University, Shanghai 200092, China. [3] Nanchang Institute of Technology.

Table S1 Detailed information of sampling sections physiochemical parameters from the Xiangjiang River.

	longitude	latitude	Depth (m)	pH	TOC (mg/L)	TN (mg/L)
S1 (Zhuzhou)	27.85523	113.08200	9.0	7.50	2.47	5.36
S2 (Zhuzhou)	27.85445	113.07267	5.5	7.32	2.28	6.04
S3 (Xiangtan)	27.85732	113.06084	6.7	7.18	2.09	9.01
S4 (Xiangtan)	27.85334	113.04751	3.9	7.28	2.20	6.49
S5 (Xiangtan)	27.84595	113.03358	3.0	7.49	2.23	9.73
S6 (Xiangtan)	27.83285	113.01888	5.9	7.46	2.41	11.66
S7 (Xiangtan)	27.81346	112.99772	6.0	7.28	2.97	15.03
S8 (Xiangtan)	27.80636	112.96767	4.3	7.57	2.57	17.88
S9 (Xiangtan)	27.79979	112.95024	2.3	7.48	2.57	13.19
S10 (Xiangtan, water-intake)	27.79740	112.93475	3.5	7.37	2.60	8.78

Table S2 Level of the potential ecological risk criteria for heavy metal.

E_r^i value	Single ecological risk	RI value	Comprehensive potential ecological risk	Reference
$E_r^i < 40$	Low	RI < 150	Low	
$40 \leqslant E_r^i < 80$	Moderate	$150 \leqslant$ RI < 300	Moderate	
$80 \leqslant E_r^i < 160$	Considerable	$300 \leqslant$ RI < 600	Considerable	[1]
$160 \leqslant E_r^i < 320$	High	$600 \leqslant$ RI	High	
$320 \leqslant E_r^i$	Very high			

Table S3 Input parameters for assessment of metal exposure and health risk.

Exposure parameter	Adult males	Adult females	Children	References
IR (L/day)	2	1.6	1	[2]
EF (days/year)	350	350	350	[2]
BW (kg)	65	55	16	[3]
ED (year)	30	30	6	[2]
AT for non-carcinogenic effect (days)	10,950	10,950	2190	[4]
AT for carcinogenic effect (days)	25,550	25,550	25,550	[4]
SA (cm^2)	19000	16000	6800	[2]
ET (h/day)	0.75	1	0.5	[4]

Table. S4 The parameters for the computation of ADD, HQ, HI and CR.

	V	Mn	Fe	Co	Ni	Zn	Sb	Ba	As	Cr	Cd	Reference
K_p	0.001	0.001	0.001	0.0004	0.0002	0.0006	0.001	0.001	0.001	0.002	0.001	[5]
$RfD_{ingestion}$ (μg/kg/day)	7	24	300	0.3	20	300	0.4	14	0.3	3	0.5	[6-8]
RfD_{dermal} (μg/kg/day)	0.182	0.96	45	0.3	0.8	60	0.06	70	0.3	0.075	0.025	[6-8]
$CSF_{ingestion}$ $(mg/kg/day)^{-1}$									1.5	0.5	6.1	[5]
CSF_{dermal} $(mg/kg/day)^{-1}$									50	500	6100	

Table S5. Heavy metal toxicity load (HMTL, μg/L) of the overlying water based on relative level of heavy metals.

Overlying water	Toxicity of heavy metals (μg/L)									
	Cr	Mn	Co	Ni	Zn	As	Cd	Sb	Ba	HMTL
S1	308.1	884.7	47.2	1075.8	7130.5	11271.1	1113.7	1140.9	24180.0	47151.9
S2	267.9	1469.1	50.6	1178.4	12002.9	11083.9	1258.7	1140.9	24690.7	53143.1
S3	209.9	80969.9	721.2	3856.2	43436.0	7606.2	1854.0	1344.2	27901.3	167898.9
S4	261.9	1637.8	64.0	4364.2	13740.7	9966.6	1188.4	1194.0	25292.0	57709.7
S5	193.5	23880.8	131.4	1666.6	15635.1	9583.9	1605.8	1204.0	26494.7	80395.8
S6	154.8	39513.9	182.0	1295.9	16336.6	9109.1	1634.3	1170.9	25676.0	95073.5
S7	147.3	36761.6	158.4	1191.6	9556.1	7472.2	1227.9	1142.9	24438.7	82096.7
S8	299.2	28038.5	215.7	1160.2	16166.2	9391.2	1858.4	1093.8	25380.0	83603.0
S9	177.1	12548.8	64.0	855.6	6678.6	8729.2	946.8	1111.9	23884.0	54995.9
S10	175.6	3832.2	57.3	979.8	8518.3	8025.2	843.5	1119.9	23414.7	46966.5
total	2195.3	229537.3	1691.7	17624.1	149200.9	92238.7	13531.5	11663.4	251352.0	769034.9
[a] Hazard intensity scere (HIS)	893	797	1011	993	913	1676	1318	601	800	
Permissible toxicity load (μg/L)	44650	239100	2022	69510	4565000	1676	3954	3606	1600000	
Removal of toxic metal to reduce pollution load	PTL	PTL	PTL	PTL	PTL	98.18%	70.78%	69.08%	PTL	

a ATSDR 2019

PTL：within permissible toxicity load.

Table S6 Non-carcinogenic Health risk via drinking ingestion of HQ and HI for adult male.

	V		Cr		Mn		Fe		Co		Ni		Zn		As		Cd		Sb		Ba		HI	
	ingestion	dermal	ingestion	dermal	ingestion	dermal	ingestion	dermal	ingestion	dermal	ingestion	dermal	ingestion	dermal	ingestion	dermal	ingestion	dermal	ingestion	dermal	ingestion	dermal	ingestion	dermal
S1-S	0.0021	0.0006	0.0037	0.0021	0.0014	0.0003	0.0006	0.0000	0.0046	0.0000	0.0016	0.0001	0.0009	0.0000	0.6494	0.0046	0.0512	0.0007	0.1368	0.0065	0.0635	0.0001	0.9159	0.0150
S2-S	0.0023	0.0006	0.0024	0.0014	0.0033	0.0006	0.0004	0.0000	0.0072	0.0000	0.0019	0.0001	0.0018	0.0000	0.6455	0.0046	0.0556	0.0008	0.1413	0.0067	0.0651	0.0001	0.9267	0.0149
S3-S	0.0022	0.0006	0.0026	0.0015	0.0608	0.0108	0.0007	0.0000	0.0364	0.0001	0.0034	0.0001	0.0028	0.0001	0.4947	0.0035	0.0691	0.0010	0.1542	0.0073	0.0687	0.0001	0.8956	0.0252
S4-S	0.0026	0.0007	0.0023	0.0013	0.0616	0.0110	0.0003	0.0000	0.0384	0.0001	0.0092	0.0003	0.0022	0.0000	0.5181	0.0037	0.0595	0.0008	0.1576	0.0075	0.0690	0.0001	0.9208	0.0256
S5-S	0.0025	0.0007	0.0020	0.0011	0.0531	0.0095	0.0006	0.0000	0.0180	0.0001	0.0026	0.0001	0.0019	0.0000	0.5424	0.0039	0.0805	0.0011	0.1452	0.0069	0.0697	0.0001	0.9186	0.0235
S6-S	0.0022	0.0006	0.0018	0.0010	0.0665	0.0119	0.0009	0.0000	0.0190	0.0001	0.0021	0.0001	0.0015	0.0000	0.5262	0.0037	0.0708	0.0010	0.1419	0.0067	0.0669	0.0001	0.8998	0.0253
S7-S	0.0033	0.0009	0.0030	0.0017	0.0668	0.0119	0.0077	0.0004	0.0229	0.0001	0.0018	0.0001	0.0014	0.0000	0.5475	0.0039	0.0591	0.0008	0.1385	0.0066	0.0650	0.0001	0.9171	0.0265
S8-S	0.0025	0.0007	0.0033	0.0019	0.0428	0.0076	0.0025	0.0001	0.0170	0.0000	0.0017	0.0001	0.0018	0.0000	0.5263	0.0038	0.0786	0.0011	0.1314	0.0062	0.0651	0.0001	0.8732	0.0217
S9-S	0.0026	0.0007	0.0036	0.0021	0.0412	0.0073	0.0031	0.0001	0.0148	0.0000	0.0015	0.0001	0.0013	0.0000	0.5578	0.0040	0.0659	0.0009	0.1357	0.0064	0.0637	0.0001	0.8912	0.0218
S10-S	0.0020	0.0005	0.0022	0.0012	0.0033	0.0006	0.0005	0.0000	0.0056	0.0000	0.0015	0.0001	0.0009	0.0000	0.4521	0.0032	0.0317	0.0005	0.1395	0.0066	0.0622	0.0001	0.7015	0.0129
S1-O	0.0022	0.0006	0.0034	0.0019	0.0014	0.0002	0.0005	0.0000	0.0046	0.0000	0.0016	0.0001	0.0008	0.0000	0.6614	0.0047	0.0499	0.0007	0.1400	0.0067	0.0637	0.0001	0.9294	0.0151
S2-O	0.0023	0.0006	0.0030	0.0017	0.0023	0.0004	0.0004	0.0000	0.0049	0.0000	0.0018	0.0001	0.0013	0.0000	0.6504	0.0046	0.0564	0.0008	0.1400	0.0067	0.0650	0.0001	0.9277	0.0150
S3-O	0.0021	0.0006	0.0023	0.0013	0.1249	0.0222	0.0007	0.0000	0.0702	0.0002	0.0057	0.0002	0.0047	0.0001	0.4463	0.0032	0.0830	0.0012	0.1650	0.0078	0.0735	0.0001	0.9783	0.0370
S4-O	0.0029	0.0008	0.0029	0.0016	0.0025	0.0005	0.0003	0.0000	0.0062	0.0000	0.0065	0.0002	0.0015	0.0000	0.5848	0.0042	0.0532	0.0008	0.1465	0.0070	0.0666	0.0001	0.8740	0.0152
S5-O	0.0025	0.0007	0.0021	0.0012	0.0368	0.0066	0.0005	0.0000	0.0128	0.0000	0.0025	0.0001	0.0017	0.0000	0.5624	0.0040	0.0719	0.0010	0.1478	0.0070	0.0698	0.0001	0.9108	0.0208
S6-O	0.0023	0.0006	0.0017	0.0010	0.0609	0.0109	0.0009	0.0000	0.0177	0.0001	0.0019	0.0001	0.0018	0.0000	0.5345	0.0038	0.0732	0.0010	0.1437	0.0068	0.0676	0.0001	0.9062	0.0244
S7-O	0.0022	0.0006	0.0016	0.0009	0.0567	0.0101	0.0008	0.0000	0.0154	0.0000	0.0018	0.0001	0.0010	0.0000	0.4385	0.0031	0.0550	0.0008	0.1403	0.0067	0.0644	0.0001	0.7776	0.0224
S8-O	0.0025	0.0007	0.0033	0.0019	0.0432	0.0077	0.0034	0.0002	0.0210	0.0001	0.0017	0.0001	0.0017	0.0000	0.5511	0.0039	0.0832	0.0012	0.1342	0.0064	0.0669	0.0001	0.9123	0.0222
S9-O	0.0023	0.0006	0.0020	0.0011	0.0194	0.0034	0.0006	0.0000	0.0062	0.0000	0.0013	0.0000	0.0007	0.0000	0.5122	0.0036	0.0424	0.0006	0.1365	0.0065	0.0629	0.0001	0.7864	0.0161
S10-O	0.0021	0.0006	0.0019	0.0011	0.0059	0.0011	0.0004	0.0000	0.0056	0.0000	0.0015	0.0001	0.0009	0.0000	0.4709	0.0034	0.0378	0.0005	0.1374	0.0065	0.0617	0.0001	0.7261	0.0133

S:surface water; O: overlying water

Table S7 Non-carcinogenic Health risk via drinking ingestion of HQ and HI for adult female.

	V		Cr		Mn		Fe		Co		Ni		Zn		As		Cd		Sb		Ba		HI	
	ingestion	dermal	ingestion	dermal	ingestion	dermal	ingestion	dermal	ingestion	dermal	ingestion	dermal	ingestion	dermal	ingestion	dermal	ingestion	dermal	ingestion	dermal	ingestion	dermal	ingestion	dermal
S1-S	0.0020	0.0008	0.0035	0.0028	0.0014	0.0003	0.0005	0.0000	0.0043	0.0000	0.0016	0.0001	0.0008	0.0000	0.6140	0.0061	0.0484	0.0097	0.1294	0.0086	0.0600	0.0001	0.8660	0.0286
S2-S	0.0022	0.0008	0.0023	0.0018	0.0031	0.0008	0.0003	0.0000	0.0068	0.0000	0.0018	0.0001	0.0017	0.0001	0.6103	0.0061	0.0525	0.0105	0.1335	0.0089	0.0616	0.0001	0.8761	0.0293
S3-S	0.0021	0.0008	0.0025	0.0020	0.0575	0.0144	0.0007	0.0000	0.0344	0.0001	0.0033	0.0002	0.0026	0.0001	0.4677	0.0047	0.0654	0.0131	0.1458	0.0097	0.0650	0.0001	0.8468	0.0452
S4-S	0.0024	0.0009	0.0022	0.0018	0.0582	0.0146	0.0003	0.0000	0.0363	0.0001	0.0087	0.0004	0.0021	0.0001	0.4899	0.0049	0.0563	0.0113	0.1490	0.0099	0.0653	0.0001	0.8706	0.0441
S5-S	0.0024	0.0009	0.0019	0.0015	0.0502	0.0125	0.0006	0.0000	0.0170	0.0001	0.0025	0.0001	0.0018	0.0001	0.5128	0.0051	0.0762	0.0152	0.1373	0.0092	0.0659	0.0001	0.8685	0.0449
S6-S	0.0021	0.0008	0.0017	0.0014	0.0629	0.0157	0.0009	0.0001	0.0180	0.0001	0.0020	0.0001	0.0014	0.0000	0.4975	0.0050	0.0669	0.0134	0.1341	0.0089	0.0632	0.0001	0.8507	0.0456
S7-S	0.0032	0.0012	0.0029	0.0023	0.0631	0.0158	0.0073	0.0005	0.0217	0.0001	0.0017	0.0001	0.0013	0.0000	0.5176	0.0052	0.0559	0.0112	0.1310	0.0087	0.0615	0.0001	0.8671	0.0452
S8-S	0.0024	0.0009	0.0031	0.0025	0.0404	0.0101	0.0024	0.0002	0.0161	0.0001	0.0016	0.0001	0.0017	0.0001	0.4976	0.0050	0.0743	0.0149	0.1243	0.0083	0.0616	0.0001	0.8256	0.0421
S9-S	0.0025	0.0010	0.0034	0.0027	0.0389	0.0097	0.0029	0.0002	0.0139	0.0001	0.0015	0.0001	0.0013	0.0000	0.5274	0.0053	0.0623	0.0125	0.1283	0.0086	0.0603	0.0001	0.8426	0.0402
S10-S	0.0019	0.0007	0.0020	0.0016	0.0031	0.0008	0.0005	0.0000	0.0053	0.0000	0.0014	0.0001	0.0009	0.0000	0.4274	0.0043	0.0299	0.0060	0.1319	0.0088	0.0589	0.0001	0.6632	0.0225
S1-O	0.0021	0.0008	0.0032	0.0026	0.0013	0.0003	0.0004	0.0000	0.0043	0.0000	0.0015	0.0001	0.0007	0.0000	0.6253	0.0063	0.0471	0.0094	0.1324	0.0088	0.0602	0.0001	0.8787	0.0285
S2-O	0.0021	0.0008	0.0028	0.0022	0.0021	0.0005	0.0004	0.0000	0.0046	0.0000	0.0017	0.0001	0.0012	0.0000	0.6149	0.0061	0.0533	0.0107	0.1324	0.0088	0.0615	0.0001	0.8771	0.0295
S3-O	0.0019	0.0007	0.0022	0.0017	0.1181	0.0295	0.0006	0.0000	0.0663	0.0003	0.0054	0.0003	0.0044	0.0001	0.4220	0.0042	0.0785	0.0157	0.1560	0.0104	0.0695	0.0001	0.9250	0.0632
S4-O	0.0027	0.0010	0.0027	0.0022	0.0024	0.0006	0.0003	0.0000	0.0059	0.0000	0.0061	0.0003	0.0014	0.0000	0.5529	0.0055	0.0503	0.0101	0.1385	0.0092	0.0630	0.0001	0.8263	0.0292
S5-O	0.0023	0.0009	0.0020	0.0016	0.0348	0.0087	0.0005	0.0000	0.0121	0.0000	0.0023	0.0001	0.0016	0.0000	0.5317	0.0053	0.0680	0.0136	0.1397	0.0093	0.0660	0.0001	0.8611	0.0398
S6-O	0.0021	0.0008	0.0016	0.0013	0.0576	0.0144	0.0008	0.0001	0.0167	0.0001	0.0018	0.0001	0.0017	0.0000	0.5054	0.0051	0.0692	0.0138	0.1359	0.0091	0.0640	0.0001	0.8568	0.0449
S7-O	0.0020	0.0008	0.0015	0.0012	0.0536	0.0134	0.0008	0.0001	0.0146	0.0001	0.0017	0.0001	0.0010	0.0000	0.4146	0.0041	0.0520	0.0104	0.1326	0.0088	0.0609	0.0001	0.7352	0.0391
S8-O	0.0023	0.0009	0.0031	0.0025	0.0409	0.0102	0.0033	0.0002	0.0198	0.0001	0.0016	0.0001	0.0016	0.0000	0.5210	0.0052	0.0787	0.0157	0.1269	0.0085	0.0632	0.0001	0.8625	0.0436
S9-O	0.0022	0.0008	0.0018	0.0015	0.0183	0.0046	0.0006	0.0000	0.0059	0.0000	0.0012	0.0001	0.0007	0.0000	0.4843	0.0048	0.0401	0.0080	0.1290	0.0086	0.0595	0.0001	0.7435	0.0286
S10-O	0.0020	0.0008	0.0018	0.0015	0.0056	0.0014	0.0004	0.0000	0.0053	0.0000	0.0014	0.0001	0.0009	0.0000	0.4452	0.0045	0.0357	0.0071	0.1299	0.0087	0.0583	0.0001	0.6865	0.0241

S:surface water; O: overlying water

Table S8 Non-carcinogenic Health risk via drinking ingestion of HQ and HI for children.

	V		Cr		Mn		Fe		Co		Ni		Zn		As		Cd		Sb		Ba		HI	
	ingestion	dermal	ingestion	dermal	ingestion	dermal	ingestion	dermal	ingestion	dermal	ingestion	dermal	ingestion	dermal	ingestion	dermal	ingestion	dermal	ingestion	dermal	ingestion	dermal	ingestion	dermal
S1-S	0.0044	0.0006	0.0074	0.0020	0.0029	0.0002	0.0011	0.0000	0.0093	0.0000	0.0034	0.0001	0.0018	0.0000	1.3192	0.0045	0.1041	0.0071	0.2779	0.0006	0.1290	0.0001	1.8605	0.0152
S2-S	0.0047	0.0006	0.0049	0.0013	0.0067	0.0006	0.0007	0.0000	0.0146	0.0000	0.0038	0.0001	0.0036	0.0000	1.3112	0.0045	0.1129	0.0077	0.2869	0.0007	0.1323	0.0001	1.8824	0.0155
S3-S	0.0045	0.0006	0.0053	0.0014	0.1235	0.0105	0.0014	0.0000	0.0739	0.0001	0.0070	0.0001	0.0056	0.0001	1.0049	0.0034	0.1404	0.0095	0.3131	0.0007	0.1396	0.0001	1.8193	0.0266
S4-S	0.0053	0.0007	0.0047	0.0013	0.1251	0.0106	0.0006	0.0000	0.0779	0.0001	0.0187	0.0003	0.0044	0.0000	1.0525	0.0036	0.1209	0.0082	0.3201	0.0007	0.1402	0.0001	1.8704	0.0257
S5-S	0.0051	0.0007	0.0041	0.0011	0.1078	0.0092	0.0012	0.0000	0.0366	0.0000	0.0053	0.0001	0.0038	0.0000	1.1017	0.0037	0.1636	0.0111	0.2949	0.0007	0.1415	0.0001	1.8658	0.0268
S6-S	0.0045	0.0006	0.0037	0.0010	0.1352	0.0115	0.0018	0.0000	0.0386	0.0001	0.0042	0.0001	0.0031	0.0000	1.0688	0.0036	0.1438	0.0098	0.2882	0.0007	0.1358	0.0001	1.8278	0.0274
S7-S	0.0068	0.0009	0.0062	0.0017	0.1357	0.0115	0.0156	0.0004	0.0466	0.0001	0.0037	0.0001	0.0028	0.0000	1.1121	0.0038	0.1201	0.0082	0.2814	0.0006	0.1321	0.0001	1.8629	0.0273
S8-S	0.0051	0.0007	0.0067	0.0018	0.0869	0.0074	0.0052	0.0001	0.0346	0.0000	0.0035	0.0001	0.0037	0.0000	1.0691	0.0036	0.1596	0.0109	0.2669	0.0006	0.1323	0.0001	1.7737	0.0253
S9-S	0.0053	0.0007	0.0073	0.0020	0.0836	0.0071	0.0062	0.0001	0.0300	0.0000	0.0031	0.0001	0.0027	0.0000	1.1330	0.0039	0.1338	0.0091	0.2757	0.0006	0.1294	0.0001	1.8103	0.0237
S10-S	0.0041	0.0005	0.0044	0.0012	0.0067	0.0006	0.0011	0.0000	0.0113	0.0000	0.0030	0.0001	0.0018	0.0000	0.9183	0.0031	0.0643	0.0044	0.2834	0.0006	0.1264	0.0001	1.4248	0.0106
S1-O	0.0045	0.0006	0.0069	0.0019	0.0028	0.0002	0.0009	0.0000	0.0093	0.0000	0.0032	0.0001	0.0016	0.0000	1.3435	0.0046	0.1013	0.0069	0.2844	0.0006	0.1294	0.0001	1.8878	0.0150
S2-O	0.0046	0.0006	0.0060	0.0016	0.0046	0.0004	0.0009	0.0000	0.0100	0.0000	0.0036	0.0001	0.0026	0.0000	1.3212	0.0045	0.1145	0.0078	0.2844	0.0006	0.1321	0.0001	1.8844	0.0158
S3-O	0.0042	0.0005	0.0047	0.0013	0.2537	0.0216	0.0014	0.0000	0.1425	0.0002	0.0116	0.0002	0.0095	0.0001	0.9066	0.0031	0.1686	0.0115	0.3351	0.0008	0.1493	0.0001	1.9872	0.0393
S4-O	0.0058	0.0008	0.0059	0.0016	0.0051	0.0004	0.0006	0.0000	0.0127	0.0000	0.0132	0.0002	0.0030	0.0000	1.1880	0.0040	0.1081	0.0073	0.2977	0.0007	0.1353	0.0001	1.7752	0.0152
S5-O	0.0050	0.0007	0.0043	0.0012	0.0748	0.0064	0.0011	0.0000	0.0260	0.0000	0.0050	0.0001	0.0034	0.0000	1.1424	0.0039	0.1460	0.0099	0.3002	0.0007	0.1418	0.0001	1.8500	0.0230
S6-O	0.0046	0.0006	0.0035	0.0009	0.1238	0.0105	0.0018	0.0000	0.0360	0.0000	0.0039	0.0001	0.0036	0.0000	1.0858	0.0037	0.1486	0.0101	0.2919	0.0007	0.1374	0.0001	1.8408	0.0268
S7-O	0.0044	0.0006	0.0033	0.0009	0.1152	0.0098	0.0016	0.0000	0.0313	0.0000	0.0036	0.0001	0.0021	0.0000	0.8906	0.0030	0.1117	0.0076	0.2849	0.0006	0.1308	0.0001	1.5795	0.0228
S8-O	0.0050	0.0007	0.0067	0.0018	0.0878	0.0075	0.0070	0.0002	0.0426	0.0001	0.0035	0.0001	0.0035	0.0000	1.1194	0.0038	0.1690	0.0115	0.2727	0.0006	0.1358	0.0001	1.8531	0.0263
S9-O	0.0047	0.0006	0.0040	0.0011	0.0393	0.0033	0.0012	0.0000	0.0127	0.0000	0.0026	0.0000	0.0015	0.0000	1.0405	0.0035	0.0861	0.0059	0.2772	0.0006	0.1278	0.0001	1.5974	0.0152
S10-O	0.0043	0.0006	0.0039	0.0011	0.0120	0.0010	0.0009	0.0000	0.0113	0.0000	0.0030	0.0001	0.0019	0.0000	0.9566	0.0033	0.0767	0.0052	0.2792	0.0006	0.1253	0.0001	1.4749	0.0119

S:surface water; O: overlying water

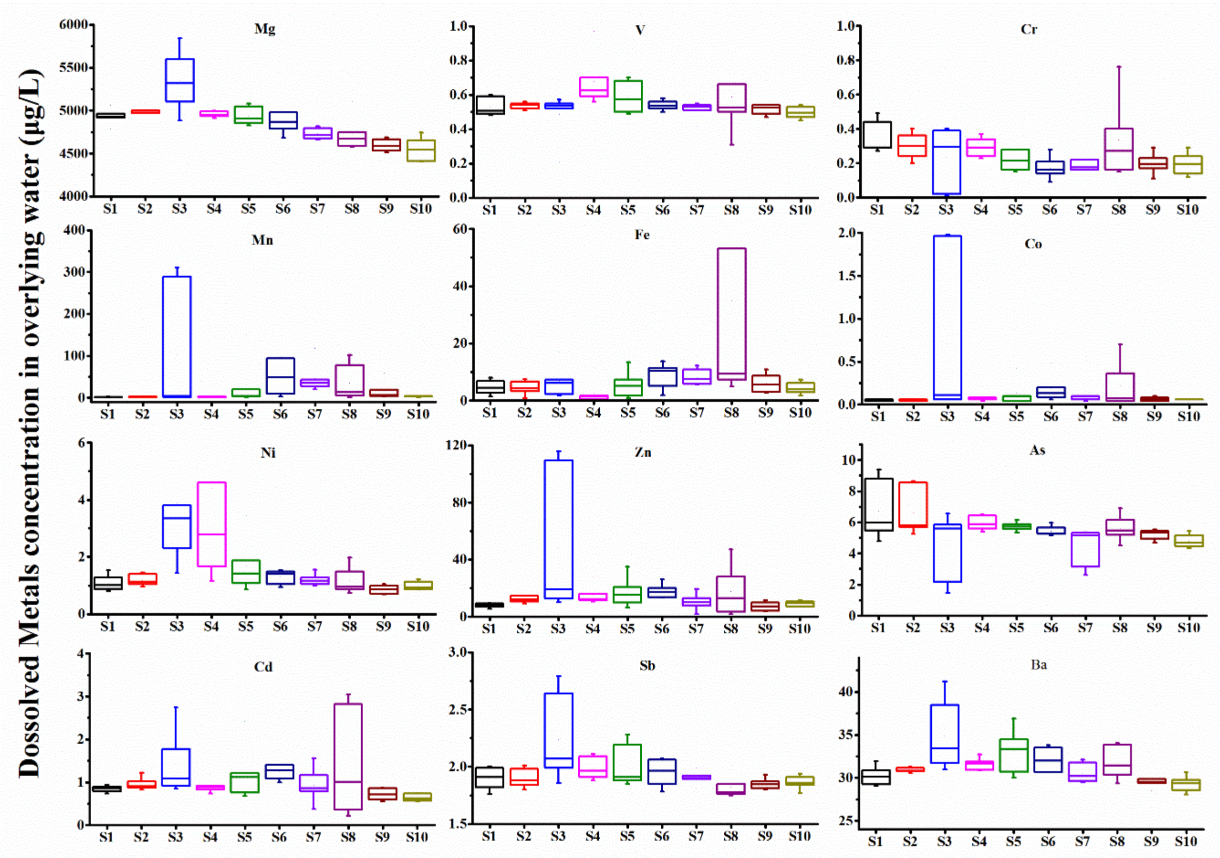

Figure S1. Spatial distribution of metals concentration in overlying water

References:

1 Ram Proshad, Tapos Kormoker & Saiful Islam. Distribution, source identification, ecological and health risks of heavy metals in surface sediments of the Rupsa River, Bangladesh. Toxin Reviews, DOI: 10.1080/15569543.2018.1564143 (2019)

2 United States Environmental Protection Agency. Risk Assessment Guidance for Superfund Volume I: Human Health Evaluation Manual (Part E, Supplemental Guidance for Dermal Risk Assessment); O_ce of Superfund Remediation and Technology Innovation: Washington, DC, USA, (2011)

3. US EPA 2012 Edition of the Drinking Water Standards and Health Advisories, Office of Water US EPA, Washington, DC. https://www.epa.gov/sites/production/files/2015-09/documents/dwstandards2012. pdf (2012).

4 United States Department of Energy (USDOE). The Risk Assessment Information System (RAIS). U.S. Department of Energy's Oak Ridge Operations Office (ORO). (2011)

5 Xu, J. et al. Identification of dissolved metal contamination of major rivers in the southeastern hilly area, China: distribution, source apportionment, and health risk assessment. Environmental science and pollution research international 27, 3908-3922, doi:10.1007/s11356-019-06774-8 (2020).

6 Yang, M. et al. Health risk assessment of groundwater pollution-a case study of typical city in North China Plain. Jour. Earth Sci. 23 (3), 335–348, (2012).

7 Wu, B. et al. Preliminary risk assessment of trace metal pollution in surface water from Yangtze River in Nanjing section, China. Bull. Environ. Contam.Toxicol. 82, 405–409, (2009).

8 Karim, Z., Risk assessment of dissolved trace metals in drinking water of Karachi, Pakistan. Bull. Environ. Contam. Toxicol. 86, 676–678, (2011).
